# Global Spatio-Temporal Patterns in Human Migration: A Complex Network Perspective

**DOI:** 10.1371/journal.pone.0053723

**Published:** 2013-01-23

**Authors:** Kyle F. Davis, Paolo D'Odorico, Francesco Laio, Luca Ridolfi

**Affiliations:** 1 Department of Environmental Sciences, University of Virginia, Charlottesville, Virginia, United States of America; 2 Department of Environmental, Land, and Infrastructure Engineering, Politecnico di Torino, Turin, Italy; University of Namur, Belgium

## Abstract

Migration is a powerful adaptive strategy for humans to navigate hardship and pursue a better quality of life. As a universal vehicle facilitating exchanges of ideas, culture, money and goods, international migration is a major contributor to globalization. Consisting of countries linked by multiple connections of human movements, global migration constitutes a network. Despite the important role of human migration in connecting various communities in different parts of the world, the topology and behavior of the international migration network and its changes through time remain poorly understood. Here we show that the global human migration network became more interconnected during the latter half of the twentieth century and that migrant destination choice partly reflects colonial and postcolonial histories, language, religion, and distances. From 1960 to 2000 we found a steady increase in network transitivity (i.e. connectivity between nodes connected to the same node), a decrease in average path length and an upward shift in degree distribution, all of which strengthened the ‘small-world’ behavior of the migration network. Furthermore, we found that distinct groups of countries preferentially interact to form migration communities based largely on historical, cultural and economic factors.

## Introduction

International migration affords distinct benefits (e.g. economic growth and poverty reduction) yet present unique challenges (e.g. human trafficking, environmental degradation, and disruption of traditions) for States and individuals alike [1]–[4]. A thorough understanding of international migration dynamics is essential to ensure that sufficient resources, services and capacities are in place so that migrants and sending and receiving countries can fully realize the prospective benefits [5] while mitigating any adverse consequences. However, adequate characterization of global human migration is lacking largely due to shortages of reliable and comprehensive global data [6], [7]. Recent studies [7], [8] were the first of their kind to construct and examine migration at the global scale using country-by-country bilateral migration data sets. The latter study showed that the number of international migrants rose from 92 million to 165 million between 1960 and 2000 and that the percent of global migration from developing to developed countries has increased markedly from 1960 to 2000. Separate work has been done on Organization for Economic Cooperation and Development (OECD) countries and on world regions, uncovering important insights regarding the interactions of migration with the political and economic sectors [9]–[11]. Other studies have also considered regional scale international migration [12] and internal migration [13]–[16].

International migration is presently occurring at unprecedented levels [8]. In 2010 the total number of people living outside their country of origin was estimated to be nearly 214 million people and projected to potentially reach 405 million people by 2050 [5], [6]. Causes and impacts of migration can be difficult to distinguish given multiple push and pull factors and often intertwined political, economic, environmental and cultural considerations [6], [17]–[19]. However, three main determinants typically dictate the specific destination, namely net benefits or improvements offered in a destination country (e.g. higher wages, health care, education), distance (be it geographical, cultural, linguistic, etc.) to that country and regulations governing immigration into that country [6], [20]. It is unclear how these determinants are mirrored in patterns and drivers of human migration and how these change over time. To date, a quantitative basis for the study of global patterns of human migration and their primary economic, socio-political, cultural or environmental drivers is missing. Here we use an approach based on complex network theory to investigate spatiotemporal patterns of international migration and hypothesize that these patterns exhibit preferential connectivity along certain country-pair links as influenced by geographical, cultural and linguistic distances over time. Recent developments in network theory [21], community structure analysis [22], [23] and available global migration data sets [7], [8] offer an opportunity for rigorous analysis of the evolution of international human movements over the latter half of the twentieth century.

The migrant populations from a given country of origin residing in a number of receiving countries form multi-directional connections. This multi-nodal system forms the global human migration network (GHMN) based on migrant stocks. Since exchanges of migrant populations between any pair of countries can occur in opposite directions in Euclidean space and thereby potentially be connected by two different links, each with a distinct magnitude, *s* (i.e. *s_ij_≠s_ji_*), the GHMN specifically constitutes a weighted directed spatial network. Thus, the global distribution of international migrants can be treated as a network of nodes (i.e., countries) connected by links representing the migrant population of country *i* living in country *j*. In this way, each node can be characterized by a degree (i.e., the total number of links connected to or from that node) and a strength (i.e., the sum of migrants who either moved from or to that node). By assembling this basic information for all nodes, the topology and behavior of the network can then be characterized through selected network metrics [24]. The most widely used and accepted of these metrics include transitivity (or clustering coefficient i.e. the probability that, if countries *a* and *b* are connected to country *c*, then *a* and *b* will connect to each other), average path length (i.e. the average shortest number of undirected connections through which a uniformly and randomly selected node *i* must travel to reach randomly selected node *j*), degree distribution (i.e. the probability that a uniformly randomly selected node will have a degree *k*) and nearest neighbor degree (i.e. the average degree of nodes directly connected to node *i*). In considering these metrics over time, we also gain insight into how processes of globalization may have potentially influenced the recent evolution of human migrations. Unlike simpler measures (e.g. total number of migrants living in each country, net migration), the analysis of network characteristics provides an integrated understanding of international human migrations and shows how changes to a node can affect the behavior and function of other seemingly unrelated nodes. Since migration occurs within a network, studying its properties is fundamental to understanding migration patterns and the underlying process of the globalization of people and cultures. In addition, this quantitative approach allows for a more comprehensive assessment of how patterns of migration have changed through time.

Supplementary to the description of migration dynamics using the network characteristics listed above, information about the connections of each node can be used to identify the community structure of the GHMN, i.e., the existence of clusters (or “communities” or “modules”) of countries characterized by overall more intense intra-community than inter-community migration. The identification of community structure is important because, by considering each module separately, it reduces the number of nodes being considered to a more manageable scale and affords the opportunity to more accurately examine the relationships and similarities causing greater intra-group interactions [25]. Communities can also be defined based on factors other than migration (e.g. common language, common religion, or population). The overlap of these communities with those of migration thus provides insight into potential influences of the migration decision. Applications of complex network theory and community identification thus present attractive analytical methods for investigating global patterns of human migration and underlying processes. Through extensive characterization of the GHMN over the last half-century (1960–2000), we establish a basis with which to examine potential drivers of migration (namely geographical, cultural and linguistic distances) and how the relationship between migration and these drivers has changed through time.

## Materials and Methods

Bilateral migration matrices of international migrant stock were used, encompassing 226 countries and territories for completed decadal census rounds centered on 1960 through 2000 (e.g. 1965 through 1974 assigned to 1970) and based on data from UN Population Division Global Migration Database and over 3500 census and population records [8]. This data set preferentially used country of birth to define country of origin; migration data are primarily provided by the destination country [8]. Former Soviet states were treated as separate throughout the decades considered, and while the connections and their magnitudes may change between decades, the countries used for each decade were held constant. For example, this means that people technically considered to be internal migrants during the years of the Soviet Union were treated as international migrants in this analysis. In this way, we eliminate the possibility of migrants suddenly being created as a result of the dissolution of a country (i.e. people remaining stationary while borders change). From the original non-symmetrical matrices, source and destination strengths (i.e. the total number of migrants that have originated from or traveled to a node, respectively) were determined for each country. Net migration is the difference between outgoing and incoming weights along a link connecting two countries, *i* and *j*.. The strength of each (undirected) link is expressed by the elements, *s*_i,j_, of a weighted matrix, calculated as the arithmetic sum of the migrants from country *i* living in *j* and of those from *j* living in *i*. To avoid double counting of connections, adjacency (i.e. 0,1) matrices derived from those of total migration for each decade were used in determining undirected country degree, *k* (i.e. the number of undirected connections between a country and its immediate network neighbors) which should not be confused with source or destination degrees calculated from the original matrices or with geographical neighbors. The undirected degree of nearest neighbor, *k_nn_*, was evaluated by:
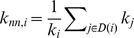
where *D(i)* constitutes the nearest neighbors of node *i* (i.e. the set of nodes directly connected to node *i*). To examine to what extent average nearest node behavior approached the maximum realizable average value for a given *k*, upper envelopes for *k_nn_* plots for each decade were calculated as:
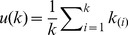
where *k_(i)_* is the vector of undirected country degree for all countries sorted in descending order [26], [27]. Average path length was determined as [28]:
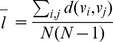
where *N* is the number of network vertices and *d(v_i_,v_j_)* is the shortest undirected network distance between vertices *i* and *j* where *i*≠*j*. Transitivity (or clustering coefficient) *C* was calculated as [21]: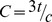 where *t* is the number of triangles of connected nodes within the network and *c* is the number of connected triples (i.e. single nodes connected to an unordered pair of other nodes). The community structure was determined as a partition of the migration network into non-overlapping communities. Community detection was based on the maximization of modularity [22], *Q*, which is defined as the following sum over all pairs of nodes
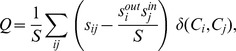
where *s*_ij_ is the weight of the link connecting nodes *i* and *j* in the net (i.e., undirected) migration network, *S* is the sum of the weights of all the network links, *s*_i_ and *s*_j_ are the strengths of nodes *i* and *j*, respectively (i.e., the sum of the weights of all links connected to *i*, and *j*, respectively), and the *δ*-function is equal to one if countries *i* and *j* are in the same community, and zero, otherwise. Thus, communities are determined by finding the partition that maximizes the modularity of the network, which is the difference between the number of intra-community links minus the number of expected connections in an equivalent network with randomly placed links [24] and expresses the strength of intra-community interaction [22]. Put simply: the greater the difference between intra- and inter-community connections, the stronger the modularity of that community. Maximization based on migrant stocks was performed for each decade using the fast greedy technique, which uses a “bottom-up” approach starting with creating small clusters of nodes that maximize the local modularity, and then iteratively aggregating these clusters until maximum modularity is attained [23]. Because of its possible sensitivity to initial conditions, the algorithm was applied one hundred times starting from different random arrangements of the nodes. If differences in community structure emerged, the partition with the highest modularity was selected [29]. Once the community structure characteristic of each decade was identified, their comparison allowed us to investigate how communities have evolved in time and the possible gradual disappearance of the legacy of old communities in the subsequent decades. Moreover, to investigate to what extent migration patterns can be explained by cultural affinities, we compare the community structure of the migrant network with those defined based on language and religion. Language based communities were defined using major colonial European languages (English, French, Spanish, and Portuguese), Russian, and Arabic. Arabic-speaking countries that were former European colonies or protectorates were placed in the Arabic community. Dominant religion was used to classify countries as Buddhist, Hindu, Christian, Islamic, or Confucian. We also investigate the community structure associated with an undirected network whose links between any pair of countries, *i* and *j*, have a weight equal to *P*_i_ *P*_j_/d_i,j_, where *P*_i_ and *P*_j_ are the populations in country *i* and *j*, respectively, while *d_i, j_* is the distance between the two countries. These weights are typically used in empirical models of social networks, known as “gravity models” [30]. Distances between geographic country centers were used to calculate the gravity-based communities. Normalized mutual information, a metric typically used to measure the interdependence between two random variables [31], was then used to compare both the community structure emerging in different decades and the community structure of the migration network with those based on language, religion, and gravity models [29].

## Results

**Figure 1:**
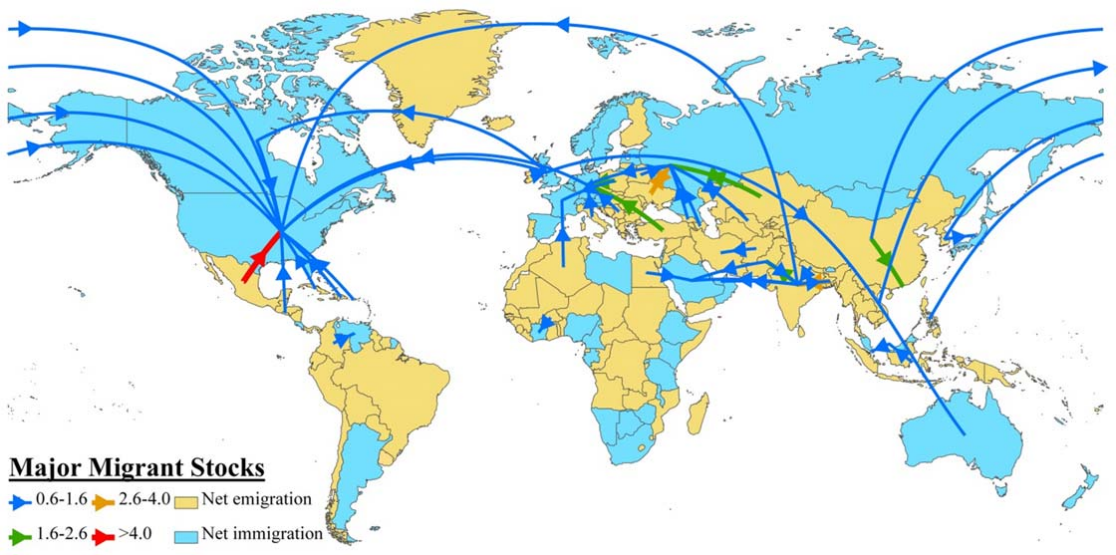
Major global migration stocks and net migration of the 2000 census. Any migrant stock of 600,000 people or more is shown. Units are in millions of migrants. Each country is designated as either a net immigration (blue) or net emigration (tan) country. The centroid of Malaysia is placed in the South China Sea between the two main halves of the country in order to make the connections from Indonesia to Malaysia and from Malaysia to Singapore visible. French Guyana is treated as a territory of France and so reflects the net migration of France.

Data for the 2000 census round (Table 1) show the largest migration connections being: 1) amongst the Middle East and India, 2) from Mexico, Canada, east Asia and western Europe to the US, 3) within Europe and 4) between Russia and neighboring eastern European and former Soviet States. The connection from Mexico to the US was the single largest with over 9 million people [8]. Overall, the international migrant stock was ∼3% relative to world population throughout the 1960–2000 period. Figure 1 also identifies each country as either a net immigration or emigration country and shows that only 34% of countries (or 76 in total) acted as net sinks of migrants, pointing toward a global tendency of many sources and fewer destinations. Interestingly, this categorization does not, however, entirely reflect North – South (i.e. developed-developing) socio-economic divisions [12].

**Table 1:** Major migration stocks for 2000 census round are shown for any link greater than 600,000 migrants.

*Source country*	*Destination country*	*Stock*	*%*	*Source country*	*Destination country*	*Stock*	*%*
Mexico	USA	9367910	5.6	India	Bangladesh	936151	0.6
Bangladesh	India	3789377	2.3	Belarus	Russia	935782	0.6
Russia	Ukraine	3613240	2.2	Uzbekistan	Russia	918037	0.5
Ukraine	Russia	3559975	2.1	South Korea	USA	896982	0.5
Kazakhstan	Russia	2584955	1.5	Cuba	USA	894560	0.5
India	Pakistan	2512906	1.5	Indonesia	Malaysia	885328	0.5
China	Hong Kong	2164744	1.3	Azerbaijan	Russia	846104	0.5
Turkey	Germany	2008979	1.2	UK	USA	833858	0.5
Poland	Germany	1999975	1.2	El Salvador	USA	827583	0.5
Russia	Kazakhstan	1931909	1.2	Poland	France	800387	0.5
Philippines	USA	1505820	0.9	Afghanistan	Iran	762129	0.5
Puerto Rico	USA	1455095	0.9	India	UAE	751142	0.4
Pakistan	India	1331659	0.8	Russia	Uzbekistan	746535	0.4
Burkina Faso	Côte d'Ivoire	1252098	0.7	Malaysia	Singapore	725607	0.4
Germany	USA	1250815	0.7	Serb. & Mont.	Germany	710269	0.4
Algeria	France	1057135	0.6	DR	USA	706894	0.4
India	USA	1041320	0.6	South Korea	Japan	685943	0.4
Vietnam	USA	1028454	0.6	Nepal	India	649166	0.4
UK	Australia	1026553	0.6	Pakistan	Saudi Arabia	638606	0.4
China	USA	1016412	0.6	Ireland	UK	636751	0.4
India	Saudi Arabia	1007649	0.6	Italy	Germany	629291	0.4
Egypt	Saudi Arabia	980205	0.6	Georgia	Russia	628973	0.4
Russia	Germany	978793	0.6	Colombia	Venezuela	617744	0.4
Canada	USA	950549	0.6	UK	Canada	606723	0.4
*Total*		49416527	29.6	*Total*		18270545	10.9
*Overall Total*						**67687072**	**40.5**

Asterisks correspond to color-coding of links in Figure 1. Abbreviations: DR = Dominican Republic, Serb. & Mont. = Serbia and Montenegro, UAE = United Arab Emirates, UK = United Kingdom, USA = United States of America

**Figure 2:**
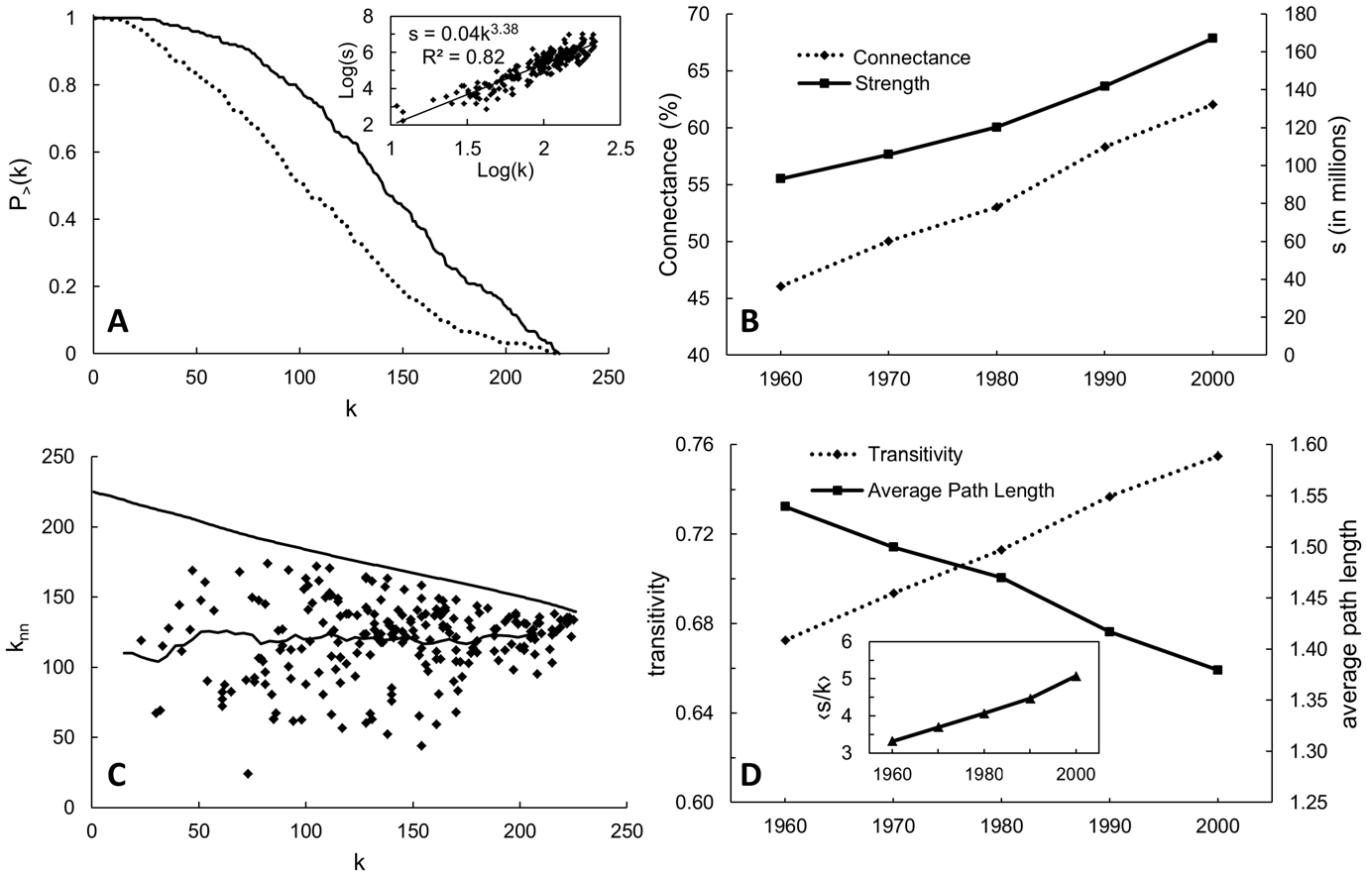
Characteristics of human migration network. (A) Cumulative undirected degree distribution of 1960 (dashed) and 2000 (solid). Plots for all other decades (not shown) progressed from the 1960 line to the 2000 line with time. The number of countries considered remains constant with time and therefore sets an upper limit on *k*. (inset of A) Log of Source strength as a function of the log of source degree for the 2000 census round. Exponent values remained consistently ∼3 for all census rounds. (B) Total strength *s* and connectance (i.e. percentage of possible undirected connections) over time. (C) Degree of nearest neighbor, k_nn_, as a function of undirected degree for each country in the 2000 census round with moving average line. (D) Network transitivity and average path length over time. (inset of D) Average source strength per source degree (thousands of people per degree) over time.

With nearly 57% of directed migration links between countries remaining throughout all decades, many key countries (e.g., USA, UK, France, India, Canada, Germany, Italy, China, Japan, Netherlands) were already highly connected in 1960, and the evolution of the GHMN in the time period examined seems largely a reflection of many countries beginning their assimilation into a globalizing world. We find this evidenced in declining percent contributions to total migration stock from the top 15 source countries from 1960 (67%) to 2000 (46%) (Table 2) and in the cumulative degree distribution (Figure 2A) where the likelihood of a randomly selected country possessing a degree greater than a certain reference value, *k*, is higher in 2000 than in any other decade considered. Transitivity increased linearly and average path length decreased linearly with time (Figure 2D) suggesting a sustained increase in direct migration connections between country-pairs that previously required the traversing of multiple links in order to reach each other. Even if countries *i* and *j* still are not connected in later decades, the addition of links to the network as a whole can indirectly reduce the shortest path length between the two countries by allowing certain intermediate links to be bypassed, and this appears to be the case. Also, transit countries have become of increasing importance as destination countries have heightened their immigration restrictions [5], therefore making average path length a more important practical measure of the GHMN. Not surprisingly, the average path length is also closest to log*N* behavior (log*N* = 2.35 vs. 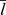 = 1.38 in 2000) which is characteristic of a non-planar spatial network [32].

**Table 2:** List of top 15 migrant source (sending) countries for 1960, 1980 and 2000 showing the number of people originating from that country (stock) and the percent of the total international migration stock for that census round.

1960			1980			2000		
*source country*	*stock*	*%*	*source country*	*stock*	*%*	*source country*	*stock*	*%*
India	9,081,881	9.8	Russia	11,682,097	9.7	Russia	10,375,787	6.2
Pakistan	8,844,720	9.5	India	7,582,096	6.3	Mexico	9,550,629	5.7
Russia	8,410,423	9.0	Ukraine	6,368,129	5.3	India	9,516,831	5.7
Ukraine	6,267,828	6.7	Bangladesh	5,047,223	4.2	Ukraine	5,915,970	3.5
Poland	5,685,110	6.1	Poland	4,800,381	4.0	China	5,814,587	3.5
China	4,803,240	5.2	Italy	4,510,364	3.8	Poland	5,147,176	3.1
Italy	4,504,270	4.8	China	4,174,988	3.5	Bangladesh	4,987,708	3.0
UK	3,507,461	3.8	UK	4,154,492	3.5	UK	4,061,775	2.4
Germany	2,734,098	2.9	Pakistan	3,970,210	3.3	Pakistan	3,812,237	2.3
Belarus	1,949,797	2.1	Germany	2,774,441	2.3	Germany	3,602,196	2.2
Spain	1,764,635	1.9	Mexico	2,579,330	2.1	Kazakhstan	3,382,369	2.0
Kazakhstan	1,328,342	1.4	Turkey	2,392,038	2.0	Italy	3,136,335	1.9
France	1,200,569	1.3	Belarus	2,319,593	1.9	Philippines	3,083,240	1.8
Czech Republic	1,186,921	1.3	Kazakhstan	2,059,098	1.7	Turkey	3,001,376	1.8
Canada	1,131,725	1.2	Spain	1,900,957	1.6	Egypt	2,267,586	1.4
*Total*	62,401,020	67.0	*Total*	66,315,437	55.2	*Total*	77,655,802	46.5

The network exhibits a power-law distribution of strength relative to degree (e.g. Figure 2A inset) where nodes with high degree have a larger stock associated with each connection. The cause of this observed behavior may be historically due to “preferential attachment” by which nodes that are newly introduced to the network have a higher probability of connecting to existent nodes that possess higher degrees [21], [33], [34]. However, the existence of this phenomenon could not be directly shown since the number of nodes is kept constant through time. In the GHMN, the power-law (*s* vs. *k*) distribution is stable with time (power law exponent ∼3 for all census rounds) and reveals: 1) that as countries increase their destination choices, the emigration population through each connection also tends to increase (Figure 2D inset) and 2) that information on degree and strength are uniquely important for characterizing the structural organization of the network [35]. The temporal increase in average nodal degree lends additional support to this evidence of increasing interconnectivity.

**Figure 3:**
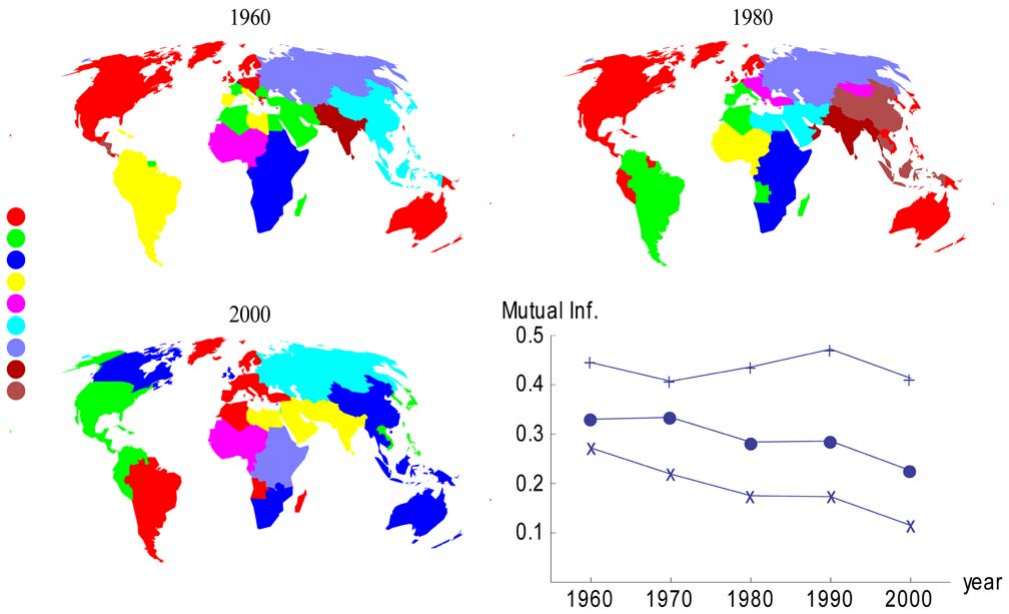
Community maps and mutual information. (A–C) The color scale indicates the strength of modularity within a community decreasing from top to bottom. As another symptom of the ongoing globalization, the global modularity of the community structures slightly decreases with time: 0.62 in 1960; 0.61 in 1970 and 1980; 0.60 in 1990; and 0.57 in 2000. Similarly, the ratio between the internal and total fluxes slowly decreases in time: 0.80 in 1960; 0.81 in 1970; 0.76 in 1980; 0.75 in 1990 and 2000. (D) The agreement between migrant communities and communities defined on the basis of religion (•), language (+) or population-based gravity models (x) was evaluated using mutual information as a measure of non-linear correlation [29].

In community analysis (Figure 3), commonalities within a community appear to be broadly founded on distance, language, religion and colonial history. Over time, this analysis shows Europe became increasingly homogeneous (Figure 3A–C), with an emergent community including most of Europe, South America (with strong migration connections with Spain and Italy), the Western Maghreb, and other former African colonies. In the last decade, the contribution of this community to the modularity of the network was higher than those of all the other communities. Over time the USA changes migration communities from northern Europe to Japan, Vietnam, the Philippines, and Caribbean countries. Canada shifts to that of the U.K. largely due to migrations from China and Southeast Asia. China has remained in the same community with other countries in Southeast Asia for the past 40 years. The Middle-Eastern community has grown over time, merging with the “Indian subcontinent” as a result of links to the Arabian Peninsula. Africa appears generally divided into North-African Arabic, Francophonic and Anglophonic communities. In 2000, southern Africa switched to the Southeast Asia-South Pacific community. Table 3 reports the mutual information between the communities of different decades. These values are consistent with the temporal evolution of the community structure shown in Figure 3a–c and express how the legacy of old communities tends to disappear in time. This tendency can be considered as a symptom of increasing globalization. Mutual information between migration communities and those defined on the basis of population-based gravity models, religion and languages (Figure 3D) indicates that religion and language explain part of the migrant community structure, though this dependence weakens over time. Between consecutive decades, mutual information remains relatively high (in the range 0.68–0.84), showing the persistence of migration patterns through time partly attributable to chain migration [20], [36].

**Table 3:** Normalized mutual information between the community structures in different decades.

-	1960	1970	1980	1990	2000
**1960**	1	0.683	0.666	0.599	0.530
**1970**	-	1	0.811	0.654	0.533
**1980**	-	-	1	0.759	0.628
**1990**	-	-	-	1	0.733
**2000**	-	-	-	-	1

## Discussion

The GHMN has increased its connectivity through the size of migrant stocks and extent of connections (Figure 2B). In the 2000 census round, 15775 connections – 62% of the possible undirected country-pair connections – had been established, the most of any decade considered. Changes in transitivity, average path length and cumulative degree distribution reveal an increasingly interconnected GHMN and point toward an enhancement of the small world effect frequently observed in complex social networks [27], [37]. Given the numerous factors that can potentially influence the rate of homogenization of the GHMN, the consistent changes in average path length and transitivity through time (Figure 2D) indicate that the interactions of migration and globalization have been persistent and stable. This means that with each time step various constructive, detrimental, intentional and unintended exchanges between countries have gained the potential to be more easily facilitated.. Given its greater extent and strength, the GHMN may be an increasingly effective and important system of vectors exerting influence on various human and natural systems.

Both the lack of dependence of *k_nn_* on undirected country degree and the high variability among countries suggest a random network behavior (i.e. neither assortative nor disassortative; Figure 2D). With no apparent relationship between *k_i_* and *k_nn_*, it seems this behavior is influenced to a certain extent by spatial constraints, meaning that while many network nodes generally have a tendency to connect to hubs, migration links of shorter geographic distance are often more cost efficient [25], [32]. This shows that the connections of a country cannot be predicted based on the degree of that country relative to others, a reasonable conclusion given the numerous factors (e.g. wage differentials, health and educational opportunities, immigration policies, language and religion) a migrant may consider in deciding to move. Community analysis reveals an overall homogenization of the GHMN over time in which larger and fewer migration communities are the eventual outcome. This is at least partially attributable to the formation of economic and political regions (e.g., European Union, Economic Community Of West African States) that facilitate international human movements preferentially between certain groups of countries [32]. The mutual information lends further support in that major cultural and demographic commonalities (e.g. religion and population) become less important in the migration decision. However, these comparisons are still important given that a number of the migration communities are not geographically contiguous, indicating that, while population and distance are generally strong determinants of migration, other factors contribute significantly to migration community structure [32]. The development of the GHMN in this way may have implications for migration resilience as well.

Throughout the evolution of the GHMN, network characteristics have developed despite underlying factors such as stricter immigration policies for many countries. Regardless of the motivations for these stricter policies (e.g. security, preservation of cultural identity, limited resource availability), this can consequently encourage potential immigrants to diversify their destination choices [6], [8]. This is supported by previous findings that internal dynamics of a migration network (e.g. duration of stay, size of migrant population) may exert greater influence on migrant movements and persist despite adverse changes to external factors (e.g. decreased wage differentials, restrictive policies) especially once a network has reached a certain threshold of maturity [16]. Many developed countries are also now beginning to realize the necessity of a large migrant work force in maintaining growth and development and in turn are making efforts to ease migration restrictions, particularly for seasonal workers and temporary migrants [9]. Supporting this notion, simulations using bilateral migration matrices have shown that an increase in allowable quotas of temporary workers (especially unskilled workers) by developed countries would increase world welfare considerably, particularly for developing countries in the form of increased remittances [7], [9], [10]. Consequently, this may modify the trajectory of future temporal dynamics in the GHMN if migrants can more easily move to the initial desired country of destination.

Keeping in mind that the undirected degrees of the most connected countries remain stable through time, the steady increase in average nodal degree across decades (Figure 2A) seems due in particular to greater migration populations and more connections to and from low and mid degree countries. One might also infer that such steady increases in emigration from a number of developing countries mirror an attainment of higher levels of human development given that median emigration rates from poor countries typically increase with human development (though rich countries understandably also display low emigration rates) [6]. This may explain the increasing involvement of certain large developing countries in the GHMN as their economies have become more globalized throughout the decades considered. If countries choose to relax their immigration policies (as described above), this relationship between migration and human development may work in the opposite direction as well in that easier migration between countries can encourage development in the source countries in the form of remittances and elevated human capital with return migration [5], [38].

The construction of the comprehensive dataset [8] used in this paper incorporated a number of simplifying assumptions which must be considered with our findings. The information on both nodal strength and degree is limited due to interpolation, propensity measures and differences in methods of reporting (citizenship vs. birth). In utilizing propensity measures, the authors of the dataset either allocated aggregated regional or global census data based on earlier or later census rounds or or divided sub-regional aggregate data based on a source country's propensity to send migrants to neighboring countries in the same destination sub-region. However, since census data was available for at least one decade for all but six countries or territories (Qatar, Eritrea, Somalia, Maldives, China and North Korea), the use of the latter type of propensity measure was far less frequently required than the former. Also, while the lack of data for these six countries might appear as a major deficiency of the data set, it only affects the statistics of migrants living in those countries because data on their emigrants were collected in the destination countries.Overall, most of our assertions about GHMN topology and behavior solely involve nodal degree and are thus largely unaffected by the issue of how accurate the magnitudes of migration strength may be. Despite the problems outlined above, most of the data for international migrant stock (91–95%) for each decade are still based on bilateral raw data or simple interpolation and therefore provide a reasonably accurate spatial and temporal picture of global migration dynamics.

Lastly, when considering the results it is essential to remember that the network analyzed here represents migrant populations (i.e. stocks) with no assertions made regarding the rate or flux of those migrants to a particular host country. Given the varied methods of census data collection and reporting employed by destination countries over time the use of fluxes can thus become problematic [8]. The stocks therefore provide an integrated picture of the migration fluxes to a country of destination (in addition to considerations such as migrant mortality, return migration and host country citizenship) in the time preceding each census round of a country. Despite these complications of analyzing stocks through time, the dataset still allows for the identification of migration communities based on a number of cultural, socio-political and economic influences. Since the data are based on decadal censuses, the time steps make the resolution of the dataset too coarse to allow identification of any transient events or processes that may have influenced migration; it may be that only longer-term and more permanent events can possibly be shown as a cause (e.g. dissolution of the Soviet Union, the partition of India or African drought). Additionally, estimates of return and irregular migrations are variable – most recently 12–37% and 10–23%, respectively [5], [6], [39] – and difficult to quantify; their potential impacts on data should thus be kept in mind with any findings.

Globalization and population growth have affected, and been affected by, various human and natural systems throughout the latter half of the twentieth century. Due to demographic, economic and technological changes, demand for migration has increased, with international migration becoming more diverse through more country-pair interactions and migrant selectivity (i.e. the tendency of better educated and more highly skilled persons to migrate) [5], [6]. Differences in modes of transportation (dependent on the distance to desired destination) as well as improvements in the affordability of and accessibility to certain forms of transportation may have impacted the dynamics of the GHMN through time, although how these differences and changes to transportation may have potentially influenced international migration is not addressed in detail in this paper. Through our analysis, increases in international migration appear to be a manifestation of such changes. We have shown that preferential migration occurs along certain connections over others, based on the interactions of numerous considerations in the migration decision, the relative importance of which is not addressed here. Specifically through the use of mutual information we have quantitatively shown that geographical, cultural and linguistic distances at least partially explain the development of global human migrations throughout the latter half of the twentieth century. Network and community analyses have therefore effectively demonstrated the overall extensification and intensification of global migration, providing a systematic basis with which to analyze any future migration data and upon which elucidations of specific migration drivers may be founded.
